# A Diagnostic Conundrum: Atypical McArdle Disease Mimic With Heterozygous PYGM Mutation and Cytochrome C Oxidase (COX)-Negative Fibers

**DOI:** 10.7759/cureus.109817

**Published:** 2026-05-28

**Authors:** Lisle W Blackbourn, Kiran K Chandrasekar, Navya Peddireddy, Gregory M Blume

**Affiliations:** 1 Neurology, University of Illinois Chicago College of Medicine Peoria, Peoria, USA; 2 Neurology, Order of Saint Francis (OSF) Illinois Neurological Institute, Peoria, USA

**Keywords:** glycogen storage disease type v, glycogen storage myopathy, mcardle disease, mcardle’s disease, metabolic myopathy, mitochondrial disorder, mitochondrial myopathy, myopathy, myophosphorylase, pygm gene mutation

## Abstract

McArdle disease is inherited in an autosomal recessive pattern, with classical disease manifesting with biallelic pathogenic variants in the PYGM (myophosphorylase) gene. Here, we present a case of a 77-year-old man with a paraspinal myopathy and head drop with cytochrome c oxidase (COX)-negative fibers who is a heterozygous carrier of a pathogenic c.148C>T (p.Arg50*) nonsense variant in PYGM as identified on a comprehensive glycogen storage disease gene panel through Mayo Clinic Laboratories. Two findings complicate a confident diagnosis of classical McArdle disease. They are explicitly addressed: a single (heterozygous) pathogenic allele for an autosomal recessive disorder, and COX-negative fibers in the absence of frank glycogen accumulation on periodic acid-Schiff staining. The case is presented as suggestive of an atypical metabolic-mitochondrial phenotype, and we discuss the diagnostic limitations of a blood-based targeted gene panel, as well as further testing, including muscle RNA sequencing and tissue-specific mitochondrial DNA analysis, that could resolve a potentially undetected second variant or a tissue-restricted mitochondrial etiology.

## Introduction

McArdle disease (glycogen storage disease type V (GSD V)) is inherited in an autosomal recessive pattern, with classical disease manifesting with biallelic pathogenic variants in the PYGM (myophosphorylase) gene [1]. This gene encodes tissue-specific muscle glycogen phosphorylase (myophosphorylase), the enzyme that initiates glycogenolysis by cleaving glycogen into glucose-1-phosphate [2]. Consequently, the fundamental pathophysiology of the disease centers on a profound intramuscular energy crisis during the early phases of exercise, as skeletal muscles are unable to mobilize stored glycogen for anaerobic and aerobic metabolism. The adult clinical presentation includes exercise intolerance, early fatigue and myalgia crises, and rhabdomyolysis in acute states marked by elevated creatine kinase or myoglobinuria [3]. Paraspinal muscle involvement and associated head drop have also been increasingly recognized as a prominent clinical manifestation of McArdle disease, particularly in older adults with MRI findings of severe fatty replacement of cervical and thoracic paraspinal muscles [4].

While symptomatic heterozygous carriers of PYGM mutations are occasionally reported, they present a diagnostic challenge as they lack the classic biallelic inactivation [5]. Furthermore, the presence of cytochrome c oxidase (COX)-negative fibers typically points toward primary mitochondrial dysfunction rather than a pure glycogenolytic defect, potentially confounding the histopathological diagnosis when classic hallmarks such as glycogen accumulation are absent [6,7].

Here, we present a case of a 77-year-old man with a paraspinal myopathy and head drop with COX-negative fibers who is a heterozygous carrier of a c.148C>T (p.Arg50*) nonsense variant in the PYGM gene (MIM:608455). This nonsense variant is an established pathogenic variant in McArdle disease [8]. These findings were somewhat suggestive of a mitochondrial process and could represent a GSD, although no frank glycogen deposits were noted on periodic acid-Schiff (PAS) stains.

## Case presentation

Case details

A right-handed man in his late seventies (full demographics, comorbidities, and family history are summarized in Table 1) presented to neurology for evaluation of progressive neck extensor weakness. He reported waking one morning unable to hold his head upright.

**Table 1:** Patient profile

Parameter	Value
Age/sex	77-year-old man
Handedness	Right-handed
Height	1.905 m (6′3″)
Weight (range over follow-up)	88.5-97.6 kg (195-215 lb)
BMI (range over follow-up)	24.4-26.9 kg/m² (within normal to overweight range)
Chronic comorbidities	Insulin-dependent type 2 diabetes mellitus (HbA1c 6.9-9.8%); atrial fibrillation (on apixaban); prostate cancer (treated with brachytherapy, in remission); hyperlipidemia (on rosuvastatin); peripheral neuropathy; essential tremor; restless legs syndrome; arthritis
Smoking history	Former smoker, 20 pack-years; quit ~40 years prior to presentation
Family history	Largely unavailable; both parents died young (father from coronary artery disease; both with chronic alcohol use). Sister reports exertional fatigue. A nephew and niece carry a diagnosis of unspecified tremor. Patient has no children
Relevant prior diagnoses	Initial outside diagnosis of cervical dystonia, treated with botulinum toxin injections with only partial, transient benefit
Presenting complaint	Subacute onset of progressive neck extensor weakness with head drop, intermittent dysarthria, intermittent dysphagia, and occasional diplopia

His primary care physician initially diagnosed cervical dystonia, and he received botulinum toxin injections with slow, partial improvement over three to four weeks; despite this intervention, the head drop persisted, worsening with activity and toward the evening. He denied any prior history of exercise intolerance, myalgia, myoglobinuria, or rhabdomyolysis.

In addition to head drop, he endorsed intermittent mild dysarthria (his wife reported episodes during which his speech was very difficult to understand), intermittent dysphagia occurring at nearly every meal, and occasional diplopia. These bulbar features raised initial clinical suspicion for myasthenia gravis (MG). The complete chronology of evaluation, intervention, and diagnostic milestones is detailed in Table 2.

**Table 2:** Diagnostic and therapeutic timeline PCP: primary care physician, MG: myasthenia gravis, MRI: magnetic resonance imaging, IVIg: intravenous immunoglobulin, NCS: nerve conduction studies, RNS: repetitive nerve stimulation, EMG: electromyography, PAS: periodic acid-Schiff, GSDGP: glycogen storage disease gene panel, TID: three times daily, RRFs: ragged-red fibers

Time point	Event	Findings/action
~1.5 years prior	Initial presentation to PCP	Acute-onset head drop diagnosed as cervical dystonia; received botulinum toxin injections with partial, transient improvement
Sep-24	Initial neurology evaluation	Persistent head drop with bulbar features (dysarthria, dysphagia, diplopia); clinical suspicion for MG; pyridostigmine 30 mg TID initiated and titrated to 60 mg TID
September–October 2024	Serologic and imaging workup	Negative AChR, MuSK, LRP4, MyoMarker 3 panel, anti-M2, anti-SRP, anti-HMGCR; CK normal. MRI brain unremarkable; MRI spine demonstrated multilevel degenerative change and moderate-to-severe paraspinal fatty atrophy. Empiric IVIg initiated for presumed seronegative MG
Oct-24	Electrodiagnostic studies	NCS within normal limits except mildly reduced ulnar conduction velocities; low-frequency RNS without significant decrement; needle EMG of cervical paraspinals myopathic, with normal recruitment in limb muscles
Nov-24	Post-IVIg follow-up	Significant clinical improvement in head holding and neck range of motion after two cycles of IVIg; residual evening/exertional worsening
Feb-25	Open muscle biopsy	Left biceps and left cervical paraspinal muscle biopsy obtained after a nine-week IVIg-free interval
Mar-25	Biopsy results reviewed	Both specimens had mild myopathic changes, sparse chronic lymphoid inflammation, mild denervation-type changes, and a moderate increase in COX-negative fibers; the paraspinal specimen had rare polyglucosan bodies and a few early RRFs; PAS without frank glycogen accumulation. Empiric ubiquinol 100 mg daily and B-complex vitamins started
Jun-25	GSDGP (Mayo Clinic Laboratories)	Heterozygous PYGM pathogenic variant identified (c.148C>T; p.Arg50*); 27 additional GSD genes negative. Genetics counseling referral made
Jul-25	Management revision	Carbohydrate loading prior to activity and a protein-rich diet adopted; pyridoxine 60–90 mg daily added; creatine supplementation considered. Power wheelchair ordered as functional status declined
Late 2025	Mitochondrial genome sequencing (peripheral blood, GeneDx)	No pathogenic, likely pathogenic, or uncertain variants identified. Note: muscle-tissue mitochondrial DNA testing recommended but not yet pursued

Neurological examination

On initial examination, the patient was alert and oriented. His head rested on his chest; he could briefly hold it upright or lift it with his hand periodically. Cranial nerve examination demonstrated possible mild right ptosis with sustained upgaze at 60 seconds, though extraocular movements were generally full and aligned. Diplopia was elicited with sustained lateral gaze, more prominent on the right. Facial musculature was symmetrically activated. Palatal rise was symmetric. The tongue was midline with normal bulk and moved freely, but repetitive tongue thrusts to either cheek demonstrated fatigable weakness. Repetitive labial and lingual sounds did not fatigue appreciably; however, guttural sounds lacked clarity and worsened with repetition. On follow-up examination two weeks later, new fatigable biceps weakness was observed bilaterally, a finding not present at the initial visit. Motor examination was otherwise notable for full power in all four extremities without pronator drift. Deep tendon reflexes were 2+ and symmetric throughout. Vibration sense was mildly diminished at the ankles bilaterally. Casual gait was unremarkable.

Laboratory/imaging investigations

An extensive serologic workup for neuromuscular junction and inflammatory myopathic disorders was uniformly negative. Acetylcholine receptor antibodies, muscle-specific kinase antibodies, and low-density lipoprotein receptor-related protein 4 antibodies were all undetectable. Serum creatine kinase levels were unremarkable. A comprehensive myositis-specific antibody panel, the MyoMarker 3 profile through Mayo Clinic Laboratories, was negative, as were anti-mitochondrial antibodies and antibodies against necrotizing myopathy, including anti-SRP Ab and anti-3-hydroxy-3-methylglutaryl coenzyme A reductase antibodies. MRI of the brain with contrast was unremarkable, except for minimal supratentorial white matter disease, likely chronic small-vessel ischemic changes. MRI of the cervicothoracic and lumbar spine showed multilevel degenerative change with multilevel neuroforaminal stenosis, severe at C3-C4 and C5-C6, and moderate-to-severe fatty atrophy of the paraspinal muscles, as seen in Figure 1.

**Figure 1:**
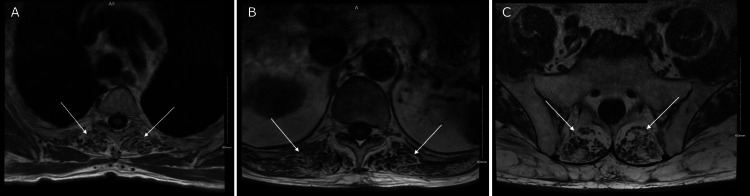
MRI cervicothoracic and lumbar spine T1-weighted images without contrast demonstrating moderate-to-severe fatty atrophy of the paraspinal muscles at various spinal levels noted in the paraspinal muscles as seen in A, B, and C MRI: magnetic resonance imaging

A dopamine transporter scan for tremors showed no evidence of presynaptic dopaminergic denervation, consistent with essential tremor rather than a Parkinsonian syndrome.

Therapeutic trial

Given the clinical suspicion for seronegative MG, a trial of pyridostigmine was initiated at 30 mg three times daily and subsequently titrated to 60 mg three times daily. The patient and his wife reported meaningful improvement in head holding and postural stability at the higher dose; no benefit was observed at 30 mg. However, pyridostigmine did not alleviate the dysphagia or diplopia. Given the progression of symptoms and the emergence of bulbar features, intravenous immunoglobulin (IVIg) was initiated empirically for presumed seronegative MG. After two cycles of IVIg, significant clinical improvement was observed: the patient was able to maintain his head upright during the clinic visit, demonstrated a full range of neck motion, and had good resistance to passive forward flexion and extension. However, the head drop still worsened with exertion, fatigue, and toward the evening. During the subsequent visit (three months after the last IVIg), head drop continued to worsen. Dysphagia improved but persisted for solid foods. On examination at that time, previously observed fatigable weakness of the biceps and tongue had resolved.

Electrodiagnostic studies

Electrodiagnostic evaluation was performed approximately four weeks after initial presentation. Of note, the patient had taken pyridostigmine earlier that morning, which may have influenced the results of repetitive nerve stimulation (RNS).

Motor nerve conduction studies (NCS) of the right ulnar nerve, recording from the first dorsal interosseous muscle, demonstrated normal distal motor latency (4.22 ms) and preserved compound muscle action potential amplitudes (10.6 mV at the wrist), with mildly reduced conduction velocities across the below-elbow (42 m/s) and above-elbow (47 m/s) segments. Bilateral facial motor NCS recordings from the nasalis muscle were within normal limits.

Low-frequency RNS at 2 Hz of the right ulnar nerve (recording at the first dorsal interosseous) and the right facial nerve (recording at the nasalis) demonstrated no significant decremental response (amplitude decrement of 4.8% and 5.8%, respectively), failing to support a neuromuscular junction defect.

Needle electromyography of the right biceps brachii and right deltoid muscles revealed no abnormal spontaneous activity, with normal motor unit action potential (MUAP) morphology and full recruitment. In contrast, needle examination of the right mid-cervical paraspinal muscles demonstrated significantly decreased MUAP amplitudes with increased polyphasia (3+), in the absence of fibrillation potentials, positive sharp waves, or fasciculations. Insertional activity was increased in this distribution. Recruitment pattern and effort were normal across all sampled muscles.

In aggregate, the electrodiagnostic findings were consistent with a myopathic process preferentially affecting the cervical paraspinal musculature, without electrophysiological evidence of a neuromuscular junction disorder or active denervation.

Muscle biopsy

Following a nine-week interval without IVIg (withheld pending tissue sampling), the patient underwent open biopsy of the left biceps (specimen A) and left cervical paraspinal muscles (specimen B).

Specimen A (left biceps) demonstrated very mild myopathic changes with sparse chronic lymphoid inflammation, mild denervation-type changes, and a moderate increase in COX-negative myofibers. Specimen B (left cervical paraspinal muscle) demonstrated very mild myopathic changes with very sparse chronic lymphoid inflammation, mild denervation-type changes, a moderate increase in COX-negative myofibers, and, notably, a few myofibers containing polyglucosan bodies. Gomori trichrome staining revealed a few early ragged-red fibers (RRFs). PAS staining did not demonstrate frank glycogen accumulation in either specimen. The background denervation-type changes consisted of occasional esterase-positive atrophic muscle fibers with a tendency toward fiber-type grouping, though fiber-type groups were not well developed. No morphologic evidence of vasculitis was identified, and changes suggestive of a chronic dystrophic process were absent. Electron microscopy was not pursued, as polyglucosan bodies were not identified in the tissue available for thick sections.

This complex combination of mild myopathic changes and sparse chronic inflammation raised the possibility of a partially sampled inflammatory myopathy with increased COX-negative myofibers. The polyglucosan bodies identified in the cervical paraspinal specimen were suspected to represent adult polyglucosan body disease. The pathologist recommended clinical correlation and, if not already performed, a myositis-specific autoantibody panel and serologic testing for anti-NT5c1A autoantibodies to further evaluate for inflammatory myopathy or sporadic inclusion body myositis. Additionally, genetic testing for polyglucosan storage disease genes (including GYG1, GBE1, RBCK1, PFKM, PRDM8, and PRKAG2) was recommended. These histopathological findings were subsequently discussed with multiple experts in neuromuscular pathology. The significance of polyglucosan bodies in paraspinal muscle, a tissue whose pathology remains incompletely understood, was uncertain. The absence of glycogen accumulation on PAS staining argued against a classical glycogen storage myopathy. The previously obtained myositis-specific antibody panel (MyoMarker) was negative; however, this panel does not include testing for anti-NT5c1A (anti-cN1A) autoantibodies, which the neuropathologist recommended to evaluate for sporadic inclusion body myositis. Nevertheless, the overall clinical phenotype, characterized by predominant axial and neck extensor weakness without the typical pattern of asymmetric distal finger flexor and knee extensor weakness, was considered atypical for sporadic inclusion body myositis.

Genetic testing

A two-stage genetic workup was performed: a comprehensive GSDGP through Mayo Clinic Laboratories, followed by mitochondrial genome sequencing of peripheral blood (GeneDx) after the unexpected detection of a heterozygous PYGM variant in the setting of COX-negative fibers and early RRFs. The methodology, specimen sources, key findings, and clinical interpretation of each assay are summarized in Table 3.

**Table 3:** Structured summary of genetic testing GSD: glycogen storage disease, NGS: next-generation sequencing, PCR: polymerase chain reaction, GSD: glycogen storage disease type V

Test performed	Methodology	Targeted genes/specimen source	Key findings/variants	Clinical interpretation and limitations
Glycogen Storage Disease Gene Panel (GSDGP, Mayo Clinic Laboratories)	NGS and/or Sanger sequencing of coding regions and intron/exon boundaries, plus PCR-based deletion/duplication analysis. Reference: GRCh37/hg19. ≥99% of bases at ≥20× depth.	28 GSD-associated genes (AGL, ALDOA, ENO3, EPM2A, FBP1, G6PC, GAA, GBE1, GYG1, GYS1, GYS2, LAMP2, LDHA, NHLRC1, PFKM, PGAM2, PGK1, PGM1, PHKA1, PHKA2, PHKB, PHKG2, PRKAG2, PYGL, PYGM, RBCK1, SLC2A2, SLC37A4). Specimen: peripheral whole blood.	Heterozygous pathogenic nonsense variant in PYGM (NM_005609.4): chr11(GRCh37):g.64527223G>A; c.148C>T; p.Arg50* (p.R50*). No additional reportable variants in the remaining 27 genes.	Established pathogenic variant associated with autosomal recessive McArdle disease (GSD V; MIM:608455). The result supports carrier status. An undetectable second variant (deep intronic, regulatory, or structural) cannot be excluded by this assay.
Mitochondrial Genome Sequencing and Deletion Testing (GeneDx)	Whole mitochondrial genome sequencing and large-scale deletion analysis.	mtDNA. Specimen: peripheral whole blood.	No pathogenic, likely pathogenic, or variants of uncertain significance identified.	A negative blood-based result does not exclude a mitochondrial etiology, as mtDNA heteroplasmy varies among tissues; muscle-tissue mtDNA testing was recommended but not yet pursued at the time of this report.

The GSDGP was performed by next-generation sequencing (NGS) and/or Sanger sequencing of coding regions and intron/exon boundaries, with PCR-based deletion and duplication analysis, against the GRCh37/hg19 reference. At least 99% of bases were covered at ≥20× depth, with reported sensitivity of >99% for single-nucleotide variants, >94% for indels under 40 bp, >95% for deletions up to 75 bp, and for insertions up to 47 bp. Within these technical bounds, the assay reliably detects exonic and near-splice-site variants but cannot resolve deep intronic or distant regulatory variants, large structural rearrangements, or mosaic events. Confirmation of a second pathogenic PYGM allele, or definitive exclusion of compound heterozygosity, therefore requires orthogonal testing. Recommended next steps include muscle RNA sequencing (RNA-seq) to identify mis-splicing or aberrant transcript signatures from deep intronic or regulatory variants undetectable on DNA-based panels; whole-genome sequencing or targeted long-read sequencing to capture structural variants in PYGM; and mitochondrial DNA sequencing on muscle tissue to address the limitation of blood-based mtDNA testing in the setting of tissue-restricted heteroplasmy.

Clinical course

Empiric supplementation with ubiquinol (coenzyme Q10, 100 mg daily) and a B-complex vitamin was initiated following the biopsy results to address the possibility of mitochondrial dysfunction. The patient and his wife reported modest improvement in stamina, though he did not regain the ability to perform new activities. He was unable to tolerate carnitine supplementation due to nausea. Following identification of the heterozygous PYGM variant, the management approach was revised to include dietary modifications consistent with McArdle disease, specifically carbohydrate loading before physical activity and a protein-rich diet. Pyridoxine (60-90 mg daily) was added, and creatine supplementation was considered for future use.

Despite these interventions, the patient experienced progressive functional decline over the ensuing months. Early in the day, he could mobilize limited distances within his home using a walker, but as the day progressed, he became wheelchair-bound. He was unable to propel the wheelchair independently due to upper back and neck weakness and no longer walked outside the home. Examination at follow-up demonstrated persistent forward flexion of the neck to the chest, with the ability to hold the head upright for progressively shorter intervals over the course of the visit. Proximal upper extremity strength was limited by the absence of stable neck and back extensor support but was near-normal when the trunk was manually stabilized. A power wheelchair was ordered.

## Discussion

McArdle disease is inherited in an autosomal recessive pattern, with classical disease manifesting with biallelic pathogenic variants in the PYGM (myophosphorylase) gene [1]. Paraspinal muscle involvement and associated head drop, as in our patient, have been increasingly recognized as a prominent clinical manifestation of McArdle disease, particularly in older adults with MRI findings of severe fatty replacement of cervical and thoracic paraspinal muscles [4]. Our patient, however, presents two findings that are not internally consistent with a confident diagnosis of classical McArdle disease and that warrant explicit reconciliation: he carries only a single (heterozygous) pathogenic PYGM variant for what is canonically a recessive disorder, and his muscle biopsy demonstrated COX-negative fibers (seen in mitochondrial myopathy) without frank glycogen accumulation on PAS staining, the histopathological hallmark of a glycogen storage myopathy.

Reconciling heterozygosity for an autosomal recessive disease

Classical McArdle disease (GSD V) requires biallelic loss-of-function variants in PYGM. Therefore, the detection of only a single pathogenic allele in our patient cannot, on its own, establish the diagnosis. Three explanations remain for the differential. First, true carrier status is most likely on a population-genetic basis: a study of seven heterozygous PYGM mutation carriers demonstrated maximal oxidative capacity and peak lactate responses identical to those of control patients, indicating that carriers as a group are not prone to developing symptoms of McArdle disease [9]. Second, an undetectable second variant on the trans allele cannot be excluded by the assay used here. The GSDGP relies on NGS and/or Sanger sequencing of coding regions and intron/exon boundaries with PCR-based deletion and duplication analysis; deep intronic variants, distant regulatory variants, complex structural rearrangements, and mosaic events fall outside its detection envelope. Notably, manifesting heterozygotes have been reported: in a study of 16 McArdle families, 14% of 50 identified heterozygous carriers reported mild myalgia or weakness. However, these carriers did not demonstrate the hallmark second-wind phenomenon or the flat lactate response to exercise seen in homozygous patients [5]. Third, a dominant-negative mechanism for selected PYGM variants has been described: a family of 13 members with a heterozygous PYGM mutation presented with adult-onset muscle weakness, in which the variant partially preserved AMP-dependent activity despite impaired AMP-independent myophosphorylase activity, leading to altered protein conformation, aggregation, and desmin accumulation in muscle fibers [10]. This represents a fundamentally novel pathophysiology, distinct from that of classic McArdle disease. Whether the c.148C>T (p.Arg50*) nonsense variant in our patient could exert a comparable effect remains speculative, as this specific allele has not, to our knowledge, been functionally characterized for dominant-negative behavior.

Broader phenotypic variability

Beyond known phenotypic boundaries, notable atypical presentations have emerged, deviating from the classic early-onset exercise intolerance associated with PYGM mutations. In one case of late presentation of McArdle disease with a heterozygous PYGM variant, a 78-year-old patient developed progressive camptocormia, or “bent spine syndrome," without any classic myalgias [11]. In another case of camptocormia, a 59-year-old female with significant exercise intolerance and a walking distance limited to 50 km was found to be positive for pathogenic variants of PYGM, c.148C > T (p.(Arg50*)) variant and a missense variant in exon 12, c.1471C > T (p.(Arg491Cys)) [12]. Clinical misattribution can be further compounded when nonspecific presentations of chronic fatigue mask metabolic etiologies until significant exertional consequences manifest [13]. Persistent elevations in creatine kinase, even without acute rhabdomyolysis, have been documented as a clinical clue that can contribute to presentations of exercise intolerance and muscle fatigue [14]. Pediatric presentations of failure to thrive and altered liver enzyme presentations have additionally been seen to obscure the diagnosis until an adult presentation of acutely severe exercise-induced rhabdomyolysis reveals a c.148C>T (p.R50X) in exon 1 and c.613G>A (p.G205S) in exon 5 heterozygous variant of PYGM [15]. Diagnosis of McArdle disease in heterozygous variants has additionally been uncovered through workup for acute musculoskeletal complaints, as seen in one case of inflammatory back pain thought to be necrotizing fasciitis until genetic testing revealed two heterozygous variants in the PYGM gene: c.1963G>A (p.Glu655Lys) class 5 and c.2178-1G>A class 4 [16]. To minimize diagnostic delays, McArdle disease must therefore be carefully evaluated within the broader context of metabolic myopathies and atypical presentations.

Reconciling COX-negative fibers with absent PAS glycogen accumulation

The biopsy phenotype is similarly unresolved. COX-negative fibers and early RRFs point toward a mitochondrial process. At the same time, the absence of frank glycogen accumulation on PAS staining argues against a classical glycogen storage myopathy of any cause, including McArdle disease, in which subsarcolemmal glycogen accumulation is the canonical histological finding. The presence of a few myofibers containing polyglucosan bodies in the cervical paraspinal specimen adds a third, separately suggestive signal. Several non-exclusive interpretations are reasonable. The biopsy may reflect a primary mitochondrial process unrelated to the PYGM finding, in which case the heterozygous PYGM variant is incidental carrier status; the negative blood-based mitochondrial sequencing does not rule this out, as mitochondrial DNA heteroplasmy varies among tissues, and muscle-specific variants may not be represented in peripheral blood. Alternatively, the COX-negative fibers may represent a nonspecific marker of fiber stress in the setting of a metabolic myopathy rather than age-related changes, with the absence of PAS-positive glycogen reflecting partial enzymatic compensation, sampling variability (paraspinal muscle pathology is incompletely characterized), or chronic myocyte loss. The polyglucosan bodies remain of uncertain significance in this anatomical distribution. We therefore frame the case as suggestive of, rather than diagnostic for, an atypical metabolic-mitochondrial phenotype with a single-allele PYGM finding of uncertain pathogenic contribution.

Diagnostic limitations and recommended further testing

Several limitations constrain interpretation. The mitochondrial sequencing was performed on peripheral blood rather than muscle, leaving tissue-restricted heteroplasmy unaddressed. Electron microscopy was not pursued. The MyoMarker panel does not include anti-NT5c1A (anti-cN1A), which the neuropathologist recommended to evaluate for sporadic inclusion body myositis, although the clinical phenotype is atypical for that diagnosis. To address the most consequential remaining ambiguity, whether a second pathogenic PYGM allele was missed by the GSDGP, we suggest that muscle RNA-seq would be the highest-yield next step, as it can detect aberrant splicing or transcript-level signatures arising from deep intronic and regulatory variants invisible to DNA-based gene panels. Whole-genome sequencing or targeted long-read sequencing would extend coverage to structural variants in PYGM, and full mitochondrial genome sequencing on muscle tissue would either implicate or substantially weaken a primary mitochondrial etiology. Pending these studies, we present the case as informative rather than conclusive: the combination of clinical phenotype, biopsy findings, and a heterozygous pathogenic PYGM variant raises questions that the current evidence cannot fully answer.

## Conclusions

We report a 77-year-old man with a paraspinal myopathy and head drop, COX-negative fibers on muscle biopsy in the absence of frank glycogen accumulation on PAS staining, and a single heterozygous pathogenic PYGM variant. Each of these findings is, in isolation, atypical of classical McArdle disease, and the combination cannot, at present, be reduced to a single unifying diagnosis. The case is best framed as suggestive of an atypical metabolic-mitochondrial phenotype rather than as a confirmed instance of McArdle disease, and it illustrates two broader lessons: that single-allele PYGM variants may, in selected patients, contribute to a clinically meaningful phenotype through mechanisms that remain incompletely characterized; and that definitive resolution of such cases will require orthogonal modalities, muscle RNA-seq, structural variant analysis, and tissue-specific mitochondrial sequencing that extend beyond the reach of standard targeted gene panels.
